# O.3.2-8 Sedentary behaviour, physical activity, and sleep patterns of women with chronic temporomandibular disorders on days with and without pain: a cross-sectional study

**DOI:** 10.1093/eurpub/ckad133.153

**Published:** 2023-09-11

**Authors:** Luiz Felipe Tavares, Luiz Augusto Brusaca, Leticia Bojikian Calixtre, Francisco Locks, Ana Beatriz Oliveira

**Affiliations:** Federal University of São Carlos, Brazil; Federal University of São Carlos, Brazil; Federal University of São Carlos, Brazil; University of Pernambuco, Petrolina, Brazil; University of Pernambuco, Petrolina, Brazil; Federal University of São Carlos, Brazil

## Abstract

**Purpose:**

Sedentary behaviour (SED) and low level of physical activity (PA) might be associated with the development or worsening of pain. Still, studies assessing physical behaviours by accelerometry in individuals with orofacial pain are limited. This study aims to assess whether women with temporomandibular disorders (TMD) present different patterns of physical behaviours in days with (DWP) or without pain (DWoP).

**Methods:**

Twenty-nine out of forty-four women (mean age 29.21 sd 7.96) were diagnosed with TMD and monitored over seven days using a thigh-worn accelerometer. DWP was determined when subjects presented pain in one of the craniocervical regions (head, jaw and neck) with intensity of at least 3 in the numerical rating scale. To be considered a DWoP, the individual presented less than 3 points in the three regions. Daily time-use compositions were described in terms of SED in short (<30 min) and long (≥30 min) bouts, light PA (LPA), moderate-to-vigorous PA (MVPA), and time-in-bed. Isometric log-ratios (ilr) were calculated to express the ratio of time-in-bed to time spent awake, SED relative to LPA and MVPA, SED in short relative to long bouts, and LPA relative to MVPA. Differences between DWP and DWoP were examined using MANOVA, followed by univariate post-hoc tests of pairwise differences.

**Results:**

During DWP, women with TMD spent more time in SED in short (239 min) and long bouts (419 min), less time in LPA (245 min), MVPA (68 min), and in bed (468 min) compared with DWoP (235, 378, 263, 70 and 493 min, respectively). The MANOVA showed that all sets of ilrs did not differ statistically (ηp2 = 0.19, p = 0.25). Still, the post-hoc tests showed a trend that time spent SED relative to LPA and MVPA was larger in DWP than in DWoP (Cohen’s d = 0.36, p = 0.05).

**Conclusions:**

Women with TMD did not show different patterns of physical behaviours in DWP or DWoP. However, there is a trend of more sedentary behaviour and less physical activity in DWP compared to DWoP. Future studies should consider other pain intensity cut-offs, isolated pain locations, and larger sample sizes to confirm these results.

